# Dissolution Profile of Mefenamic Acid Solid Dosage Forms in Two Compendial and Biorelevant (FaSSIF) Media

**DOI:** 10.3797/scipharm.ISP.2015.09

**Published:** 2016-02-14

**Authors:** Wilda Nurhikmah, Yeyet Cahyati Sumirtapura, Jessie Sofia Pamudji

**Affiliations:** School of Pharmacy Bandung Institute of Technology, Ganesha 10, 40132, Bandung, Indonesia

**Keywords:** Mefenamic acid, Dissolution Testing, Indonesian Pharmacopoeia, United States Pharmacopoeia, Pharmacopoeia of the People’s Republic of China, Biorelevant medium

## Abstract

Mefenamic acid is a non-steroidal anti-inflammatory drug (NSAID) that is widely used for the treatment of mild-to-moderate pain. Mefenamic acid belongs to the Biopharmaceutical Classification System (BCS) class II drug which has lower water solubility but high permeability. There are two different compendial methods available for dissolution tests of mefenamic acid solid dosage forms, i.e. methods of United States Pharmacopeia 37 (USP) and Pharmacopoeia of the People’s Republic of China 2010 (PPRC). Indonesian Pharmacopeia V ed. (FI) adopted the USP method. On the other hand, many researches focused on the use of a ‘biorelevant’ medium to develop the dissolution test method. The aim of this research was to study the dissolution profile of mefenamic acid from its solid dosage forms (caplet and capsule) available in the Indonesian market with three different dissolution medium: USP, PPRC, and biorelevant fasted simulated small intestinal fluid (FaSSIF) media. The tested products consisted of the innovator’s product (available only in caplet dosage form, FN caplet) and generic products (available as caplet and capsule). The dissolution test of the drug products in all dissolution media was performed in 900 mL of medium using apparatus II (paddle) at a temperature of 37°C and rotation speed of 75 rpm, except for the capsule product and for USP medium, both of which tests were done using apparatus I (basket) with rotation speed of 100 rpm. The solubility test of mefenamic acid was carried out in all media at temperature of 37°C. The result obtained from the solubility test showed that the the highest solubility of mefenamic acid was obtained in USP medium (approximately 2 mg/mL), followed by PPRC medium (about 0.5 mg/mL), and FaSSIF medium (approximately 0.06 mg/ml). In the dissolution test, percentage of drug dissolved in in the USP and PPRC media after 45 min for all products reached more than 75%, except for the PN caplet in USP medium which reached only about 44%. Meanwhile, in the biorelevant medium, the percentage of drug dissolved for all products did not exceed 16%. In all dissolution media, the capsule dosage form achieved the highest dissolution rate.

## Introduction

Dissolution is generally defined as a process by which a solid substance is solubilized into the solvent to yield a solution. Drug dissolution characteristic is an important quality parameter to ensure in vivo performance of solid drug products, especially for generic drug products since the drug absorption process from solid dosage forms (especially for drugs given by oral route) depends on the release of the drug substance from the drug product, the dissolution or solubilization of the drug under physiological conditions, and the permeability across the gastrointestinal tract. Because of the critical nature of the first two mentioned steps, in vitro dissolution may be relevant to the prediction of in vivo performance.

Mefenamic acid [2-[(2,3-dimethylphenyl)amino]benzoic acid], an anthranilic acid derivative, is a non-steroidal anti-inflammatory drug (NSAID) which is widely used to relief mild to moderate pain [1]. Mefenamic acid is classified as class II on the basis of biopharmaceutical classification system (BCS). It has low water solubility but high permeability. The absolute bioavailability of this drug is about 90–100% [2]. For BCS class II drugs, the dissolution process is critical in ensuring high or acceptable drug absorption. Mefenamic acid is widely used in Indonesia and available as solid dosage form for oral administration (capsule and caplet).

Currently, there are two approved compendial dissolution testing methods available for quality evaluation of mefenamic acid solid dosage forms. The first method is mefenamic acid dissolution test stated in the United States Pharmacopeia 37 (USP 37) and the other method is the one stated in Pharmacopoeia of the People’s Republic of China 2010 (PPRC 2010). Meanwhile, Indonesian Pharmacopoeia 5th edition (FI V) adopts the method of USP. There are some differences between USP method and PPRC method.

USP provides the dissolution test of mefenamic acid solid dosage form only for capsule. In this test, USP urges the application of type 1 (basket) dissolution test apparatus set at rotation speed of 100 rpm, using 900 mL tris buffer at pH 9 containing sodium lauryl sulfate 1% as dissolution medium. The stated acceptance criterion is that not less than 75% *(Q)* of the labeled amount of mefenamic acid is dissolved after 45 min [3].

PPRC states dissolution test for both mefenamic acid capsule and caplet/tablet. PPRC uses apparatus type 2 (paddle) with the rotation speed set at 75 rpm and with 800 ml phosphate buffer pH 8 which contains 40 mL ethanol as the dissolution medium. The acceptance criteria for mefenamic acid in capsule and capsule dosage forms are stated not less than 70% *(Q)* and not less than 60% *(Q)* of the labeled amount of mefenamic acid is dissolved in 45 min, respectively [4].

Indonesian Pharmacopoeia 5th edition (FI V) adopts USP for quality requirements of mefenamic acid solid dosage forms [5]. Accordingly, only mefenamic acid capsule dosage form which should be tested for dissolution characteristic, while in tablet/caplet dosage form, the disintegration test replaces the dissolution test. This situation is not favorable for drug that belongs to BCS class 2 owing to the rate of dissolution is the major factor that limits the absorption process of such drug. Moreover, at this moment, generic mefenamic acid (solid oral) dosage forms in Indonesia are not listed in drugs required to undergo bioequivalence test.

Many recent researches focus on the use of ‘biorelevant’ medium to develop dissolution test method. The biorelevant medium is the medium which simulates human intestinal fluid or gastric fluid. The biorelevant mediums are developed by Jennifer B Dressman in 1998 which consist of simulated human intestinal fluid and simulated gastric fluid [6-8]. The simulated human intestinal fluids comprise both fasted state simulated small intestinal fluid (FaSSIF) and fed state simulated small intestinal fluid (FeSSIF). Meanwhile, the simulated gastric fluids consist of fasted state simulated gastric fluid (FaSSGF) and fed state simulated gastric fluid (FeSSGF). This biorelevant medium contains natural surfactant from human intestine, i.e. sodium taurocholate and lecithin.

The aim of this research was to study the dissolution profile of mefenamic acid from solid dosage forms which are commercially available in Indonesian market with three different methods/mediums, i.e. the USP method, PPRC method and using the fasted state simulated intestinal fluid (FaSSIF) biorelevant medium, in order to elucidate the optimal dissolution test condition which is the most appropriate/suitable for mefenamic acid tablet dosage form.

## Results and Discussion

### Solubility test

**Fig. 1:**
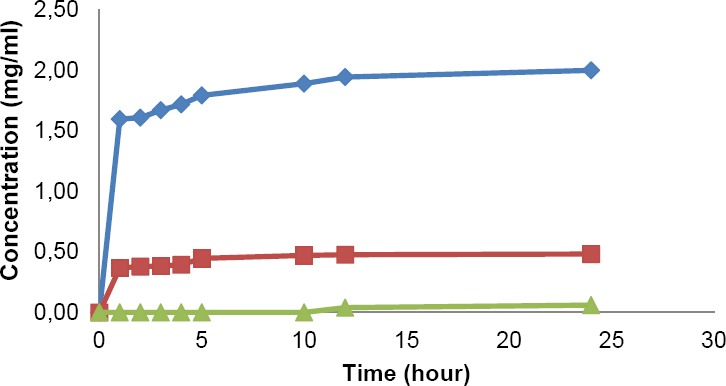
Solubility test results in USP 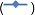, PPRC 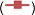 and FaSSIF 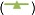 medium.

The result obtained from solubility test (presented in Fig 1) showed that the highest solubility of mefenamic acid was attained in USP medium (about 2 mg/mL). Solubility in USP medium was approximately 31 fold higher than that of FaSSIF medium (0.06 mg/mL) and 4 fold higher than that of PPRC medium (circa 0.5 mg/mL). Based on these data, USP and PPRC dissolution medium with a volume of 900 mL have the highest capacity to dissolve high dose (500 mg) of the drug (mefenamic acid) up to 100%. Meanwhile, FaSSIF medium is not entirely suitable for the dissolution test of the drug as mefenamic acid solubility in this medium is very low.

Solubility profiles of mefenamic acid in several solvents and dissolution medium have been published earlier and some results differ significantly from one to another [9, 10]. Mefenamic acid has poor solubility in water of which range spanned from approximately 0,004 mg/mL (37 °C [9]) to 0.2 mg/mL (25 °C [10]). On the other hand, mefenamic acid has high solubility in water-miscible organic solvent, namely ethanol (14.8 mg/mL at 25 °C [10]) and polyethylene glycol 400 (11.5 mg/mL at 25 °C [10]). The fact that the solubility of the drug is high in water-miscible organic solvent is used as co-solvent for mefenamic acid dissolution medium in PPRC. The addition of some hydrophilic surfactants, such as Brij-35, sodium lauryl sulphate (SLS), and Tween 80, increased correspondingly the solubility of the drug in water. The use of SLS in concentration of 2–10% increased the solubility of the drug up to 0.852 mg/mL [9, 10]. Beside, pH significantly altered the solubility of mefenamic acid in water. Being an acidic drug, mefenamic acid solubility in water was raised by the increasing of pH. At pH of 9.0, the solubility of the drug at 37°C could reach more than 0.1 mg/mL [Swathi]. The USP method is benefitted by this pH effect and further supported by the addition of SLS for the dissolution medium of mefenamic acid capsule in its monograph [3].

### Dissolution test

**Fig. 2:**
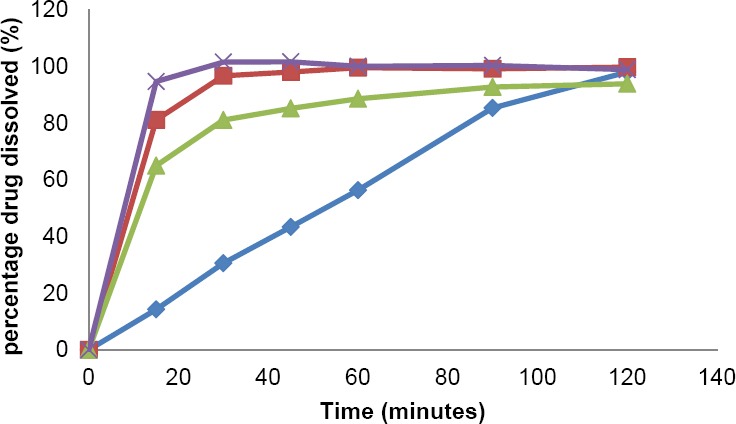
Mefenamic acid dissolution profile obtained with USP method for PN Caplet 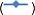, ML Caplet 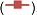, GN Caplet 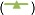 and ML Capsule 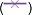

The dissolution profile in USP medium is depicted in Figure 2 and the data is detailed in Table 1. In USP 37, the dissolution requirement for mefenamic acid solid dosage form is provided only for mefenamic acid capsule, as described in its monograph. Acceptance criteria for mefenamic acid capsule in USP 37 is stated as not less than 75% *(Q)* of the labeled amount of mefenamic acid is dissolved after 45 min. The results showed that ML capsule met the requirement, where each unit yielded percent of drug dissolved after 45 min that were not less than 80%, i.e. the average value was 101.68 ± 1.51 %. GN and ML caplet also showed the possibility to meet the requirement, of which average percentage of drug dissolved in 45 min was not less than 75%. Meanwhile, innovator’s caplet product (FN 500) showed average (from 12 units) percentage of mefenamic acid dissolved in USP medium after 45 min that were less than 50% (43.37 ± 8.34 %). Based on this result, USP dissolution requirement for mefenamic acid in capsule dosage form cannot be translated or applied directly for its caplet dosage form.

**Tab. 1:**
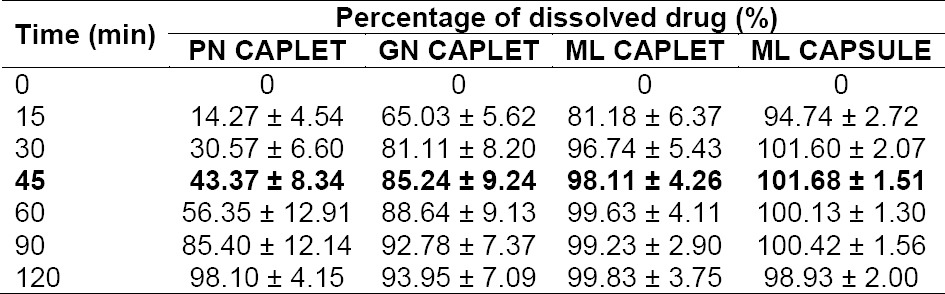
Percentage of drug dissolved in USP medium

**Fig. 3:**
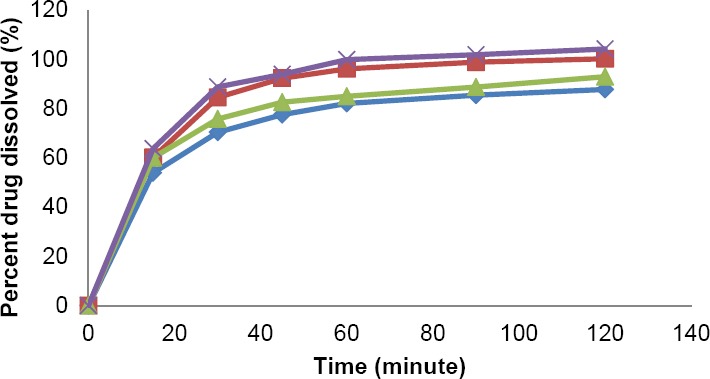
Mefenamic acid dissolution profile using PPRC medium for PN Caplet 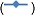, GN Caplet 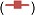, ML Caplet 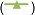 and ML Capsule 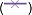.

The dissolution profiles of mefenamic acid and its data in PPRC medium are portrayed in Figure 3 and Table 2, respectively. Acceptance criteria for mefenamic acid capsule in PPRC is that not less than 70% *(Q)* of the labeled amount of mefenamic acid is dissolved in 45 min, whereas for caplet dosage form is stated as not less than 60% *(Q)* of the labeled amount of mefenamic acid is dissolved in 45 min. The results showed that all caplet and capsule products could meet the requirement. PN caplet showed the least of dissolved drug (77.55% ± 5.02%), followed by ML caplet (82.62% ± 1.36%), GN caplet (90.96% ± 3.28%), and ML capsule (93.97 % ± 10.33%) in decrease order of magnitude. ML capsule showed the highest percentage of dissolved drug due to it contained only 250 mg of the drug, which are lower by half than that in caplet dosage form, i.e. 500 mg. In PPRC medium, all mefenamic acid in capsule dosage form with dosage strength of 250 mg could be dissolved completely.

**Tab. 2:**
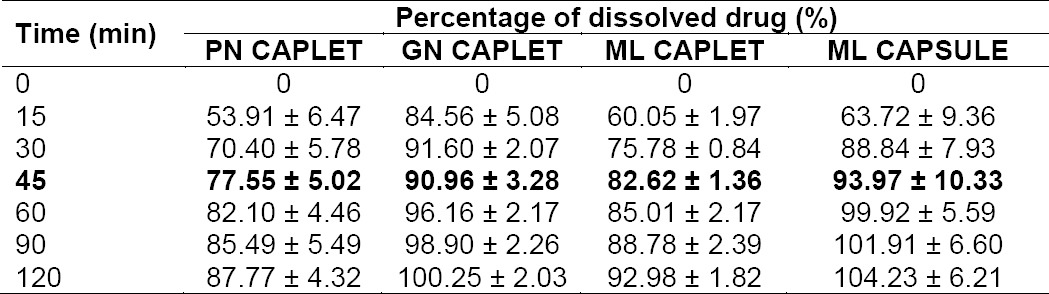
Percentage of drug dissolved by using PPRC method

In FaSSIF medium, percentage of drug dissolved in all tested products in 45 min were not more than 16%. The highest percentage of drug dissolved in this medium that was also achieved by capsule dosage form was likely as a result of the same cause as in the other mediums. This result was in good agreement with those obtained by other researchers previously [8–11]. Considering the absolute bioavailability and the pharmacokinetic profile of mefenamic acid, it seemed that in this case the use of biorelevant dissolution medium (with USP dissolution apparatus) would not able to reflect the in vivo performance of mefenamic acid. Mefenamic acid from PN capsule or caplet has a good bioavailability (90–100%) with time to achieve maximum concentration in plasma ranged from 2 to 4 hours [1, 2].

**Tab. 4:**
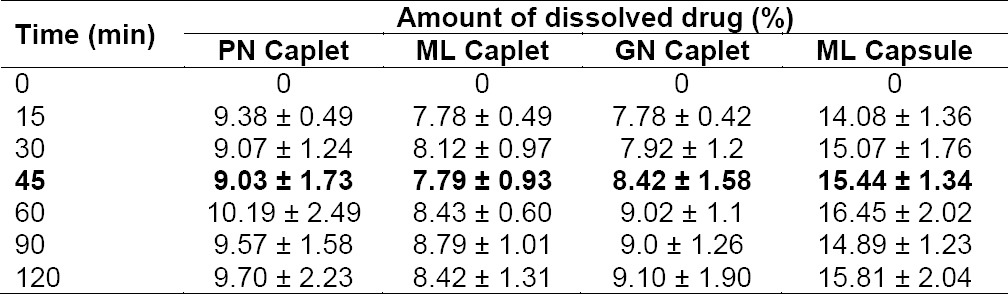
Percentage of drug dissolved in FaSSIF medium with USP apparatus

**Fig. 4:**
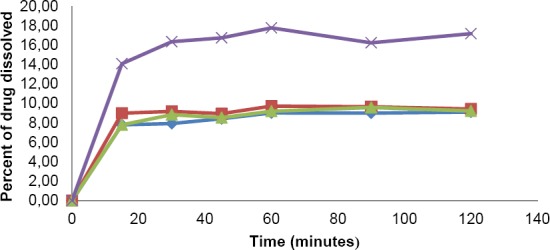
Mefenamic acid dissolution profile using FaSSIF medium for PN Caplet 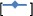, GN Caplet 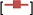, ML Caplet 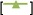 and ML Capsule 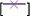.

## Results

From all results obtained in this study, it could be seen that the dissolution test method of PPRC can be choosen for dissolution test of both capsule and caplet mefenamic acid dosage forms. The USP method and requirement for mefenamic acid capsule dosage form could not be applied for caplet dosage form since the percentage of drug dissolved in 45 min for the latter dosage form (innovator’s product) was very low (below 50% and could not meet the requirement).

The medium in USP method might be applied for compendial dissolution requirement for mefenamic acid caplet owing to the high enough solubility of mefenamic acid in this medium to obtain percentage of drug dissolved more than 80% (for 500 mg dosage strength). In this study, the percentage of drug dissolved from innovator’s caplet product in this medium that was more than 80% was reached at dissolution time of 90 min. To obtain this required percentage faster, the condition related to the dissolution apparatus could be modified, i.e. using type 2 apparatus (paddle) with relatively high rotation speed (75 or 100 rpm). If the apparatus condition will be maintained the same (not modified), the requirement can be changed instead, for example: not less than 80% of drug dissolved at sampling time of 90 min. In fact, by using the same dissolution condition as for capsule product, other mefenamic acid caplet products (generic products) could release the active ingredient more than 80% in 45 min. However, in this case, the dissolution characteristic of the innovator’s product could be possibly concluded as not meet the dissolution test acceptance criteria. It should be kept in mind that this should be considered as merely the failure to fulfill the dissolution (in vitro) requirement and not to justify that the innovator’s product in vivo performance is inferior. In vitro dissolution requirement is applied to ensure and roughly predict the in vivo performance of drug products, while innovator’s product is the drug product which has been fully evaluated for its safety and efficacy.

Other alternative which is also possible to be applied is by using surfactant which can increase the solubility of the drug significantly in physiological pH range, such as cetyl-trimethylammonium-bromide (CTAB) or cetrimide. Some studies have found that the addition of cetrimide could raise the solubility and dissolution rate of mefenamic acid to a far higher extent than those resulted by other surfactants, such as Tween 80 and SLS [12, 13]. A study showed that the use of 1% CTAB in 900 mL of phosphate buffer at pH 6.8 could result in more than 80% mefenamic acid release from the 500-mg caplet after 30 min of dissolution time [13].

In all cases, in vitro-in vivo correlation should ideally be tested in order to obtain plausible prediction of in vivo performance of the drug.

## Conclusion

The result obtained from solubility test showed that the best solubility of mefenamic acid was in USP medium (approximately 2 mg/mL), followed by the solubility in PPRC medium (about 0.5 mg/mL) and in FaSSIF medium (about 0.06 mg/mL). In the dissolution test, percentage of drug dissolved in both USP and PPRC medium after 45 min for all products reached more than 75%, except for PN caplet which reached only about 44% in USP medium. Meanwhile, the percentage of drug dissolved for all products in biorelevant medium were not more than 16%. In all of the dissolution mediums, the highest dissolution rate was achieved by capsule dosage form. All of the tested products could meet the USP and PPRC dissolution requirements. USP dissolution requirement for capsule dosage form could not be applied for the requirement of caplet product.

## Experimental

### Material

Mefenamic acid was purchased from Zhejiang Qiming Pharmaceutical Co., Ltd. The commercial products of mefenamic acid which were tested were Ponstan® 500-mg caplet batch number (BN) 3 0225 B (abbreviated as PN), branded generic (Mefinal® 500-mg caplet BN RC 8639 and 250-mg capsule BN RB 2042, abbreviated as ML), and non-branded generic 500-mg caplet BN 502251 produced by PT Hexpham Jaya (abbreviated as GN), all of which were sampled from Indonesian market. FaSSIF biorelevant medium was purchased from Biorelevant.com Ltd, Croydon, Surrey, UK. All other chemicals and reagents were of analytical grades.

### Methods

#### Solubility test

Solubility test of mefenamic acid was performed in 10 mL of medium by using orbital shaker set at the temperature of 37°C and rotation speed of 100 rpm. Samples were withdrawn at 1, 2, 3, 4, 8, 12, 24 and 48 h.

#### Dissolution test

All dissolution tests used 12 units of each drug product, except for those in FaSSIF medium (only 4 units). The dissolution test of the drug products in all dissolution medium was performed in 900 mL of medium using apparatus II (paddle) at temperature of 37°C and rotation speed of 75 rpm, except for capsule product and for USP medium, both of which the test were done using apparatus I (basket) with rotation speed of 100 rpm. In all of the experiments, 5 mL of dissolution medium samples were withdrawn at 15, 30, 45, 60, 90, and 120 min. The replacement with an equal volume of the fresh medium to maintain a constant total dissolution medium volume was carried out after each time of sampling. Samples taken from the dissolution medium were filtered immediately, diluted with corresponding dissolution medium if necessary and assayed by UV spectrophotometry method. Cumulative percentages of the drug dissolved from the tested products were calculated by taking into account the samples withdrawn previously.

#### Drug assay

Drug concentrations in the solubility and dissolution tests were determined by UV spectrophotometry at the wavelength of 285 nm for samples taken from USP and PPRC medium and at 280 nm for those from biorelevant medium, in accordance with the maximum wavelength of mefenamic acid in each dissolution mediums. The assay was verified before it was used for the drug assay.

## Authors’ Statement

### Competing Interests

The authors declare no conflict of interest.
